# Toll-Like Receptor-4 Dependent Intestinal and Systemic Sequelae Following Peroral *Campylobacter coli* Infection of IL10 Deficient Mice Harboring a Human Gut Microbiota

**DOI:** 10.3390/pathogens9050386

**Published:** 2020-05-18

**Authors:** Sigri Kløve, Claudia Genger, Soraya Mousavi, Dennis Weschka, Stefan Bereswill, Markus M. Heimesaat

**Affiliations:** Institute of Microbiology, Infectious Diseases and Immunology, Charité - University Medicine Berlin, Corporate Member of Freie Universität Berlin, Humboldt-Universität zu Berlin, and Berlin Institute of Health, 12203 Berlin, Germany; sigri.klove@charite.de (S.K.); claudia.genger@charite.de (C.G.); soraya.mousavi@charite.de (S.M.); dennis.weschka@charite.de (D.W.); stefan.bereswill@charite.de (S.B.)

**Keywords:** Toll-like Receptor-4, lipooligosaccharide, *Campylobacter coli*, campylobacteriosis model, host–pathogen interaction, human microbiota-associated IL-10^-/-^ mice, pro-inflammatory immune responses, intestinal immunopathology, systemic immune responses, human gut microbiota, fecal microbiota transplantation

## Abstract

Zoonotic *Campylobacter*, including *C. jejuni* and *C. coli*, are among the most prevalent agents of food-borne enteritis worldwide. The immunopathological sequelae of campylobacteriosis are caused by Toll-like Receptor-4 (TLR4)-dependent host immune responses, induced by bacterial lipooligosaccharide (LOS). In order to investigate *C. coli*-host interactions, including the roles of the human gut microbiota and TLR4, upon infection, we applied a clinical acute campylobacteriosis model, and subjected secondary abiotic, TLR4-deficient IL10^-/-^ mice and IL10^-/-^ controls to fecal microbiota transplantation derived from human donors by gavage, before peroral *C. coli* challenge. Until day 21 post-infection, *C. coli* could stably colonize the gastrointestinal tract of human microbiota-associated (hma) mice of either genotype. TLR4-deficient IL10^-/-^ mice, however, displayed less severe clinical signs of infection, that were accompanied by less distinct apoptotic epithelial cell and innate as well as adaptive immune cell responses in the colon, as compared to IL10^-/-^ counterparts. Furthermore, *C. coli* infected IL10^-/-^, as opposed to TLR4-deficient IL10^-/-^, mice displayed increased pro-inflammatory cytokine concentrations in intestinal and, strikingly, systemic compartments. We conclude that pathogenic LOS might play an important role in inducing TLR4-dependent host immune responses upon *C. coli* infection, which needs to be further addressed in more detail.

## 1. Introduction

Campylobacteriosis constitutes an infectious syndrome caused by *Campylobacter* species, most commonly by *C. jejuni*, and less frequently by *C. coli*. Both species are considered to be among the main causative factors of bacterial gastroenteritis in humans worldwide [1]. The World Health Organization estimates that 550 million people fall ill on a yearly basis, of which 220 million are children [2]. Nonetheless, the number of incidents per year is believed to be underreported in developing countries, given that cultural confirmation remains challenging [1]. In the annual report of the European Centre for Disease Prevention and Control (ECDC) from 2018, campylobacteriosis has been stated as the most frequent gastrointestinal bacterial infectious disease in the European Union (EU) since 2005. Currently, infection with *Campylobacter* is mandatory to report in 21 EU member states. Only 55.4% of the confirmed cases provided information about the *Campylobacter* species. Of these, 83.9% were caused by *C. jejuni*, and 10.3% by *C. coli* [3]. 

The majority of the campylobacteriosis outbreaks are food-borne, due to the fact that *Campylobacter* are part of the gut microbiota of many domestic and wild animals. Raw or undercooked meat from cattle, pigs, sheep, and particularly from broiler, are sources of *Campylobacter* transmission to humans [4]. With a body temperature of above 40 °C, broilers get easily colonized by thermophilic *Campylobacter* species such as *C. jejuni* and *C. coli*. During the slaughter process, the broiler meat becomes contaminated by feces, and *Campylobacter* can survive particularly well in the feather follicles of the broiler skin [4]. Out of the speciated *Campylobacter* samples taken from broiler meat in the EU in 2018, 62.6% were reported as being contaminated with *C. jejuni*, and the remaining 37.4% with *C. coli* [3]. Although *C. jejuni* and *C. coli* share many reservoirs, their prevalence differs greatly. In sheep and pig meat, for instance, most *Campylobacter* isolates have been identified as *C. coli* [4].

After an incubation period of 1 to 5 days, patients infected by *C. jejuni* or *C. coli* present with acute watery or even inflammatory and bloody diarrhea, abdominal cramps and fever [5,6]. The disease may last for up to 10 days, is mostly self-limited and usually requires, if any, symptomatic therapy, such as rehydration and substitution of electrolytes. Antimicrobial therapy, however, might only be required in severe cases, like in infected immunocompromised patients [6,7]. In rare instances, post-infectious sequelae, such as the autoimmune neuropathies Guillain–Barré syndrome (GBS) and Miller Fisher syndrome, reactive arthritis or intestinal inflammatory morbidities, comprising irritable bowel syndrome and coeliac disease, might arise [6,8]. Moreover, there is increasing evidence linking campylobacteriosis to the occurrence of inflammatory bowel diseases (IBD) [9]. 

The host is constantly exposed to an extensive range of microbes, of which some, like *Campylobacter* spp., are potentially harmful and can cause infection [10]. In defense, the host develops complex protective mechanisms, such as the innate and adaptive immune systems. The innate immunity represents the primary line of defense, and consists of distinct cell populations, e.g., macrophages, natural killer cells and mucosal epithelial cells expressing a wide range of pattern recognition receptors (PRRs) [10]. One key PRR family is the Toll-like Receptors (TLRs), which recognize different conserved microbial ligands. Gram-negative bacteria are mainly sensed by TLR4, which binds the major cell wall components lipopolysaccharide (LPS) or lipooligosaccharide (LOS), both of which consist of a core oligosaccharide and a lipid A moiety [11,12]. LOS lacks the prolonged O-antigens found in LPS, but lipid A is nevertheless responsible for most of the immunostimulatory effects [13,14]. We and others have previously demonstrated that the intestinal and extra-intestinal—including systemic—immunopathological sequelae, upon murine *C. jejuni* infection, are mediated through LOS-induced and TLR4-dependent host immune responses [15,16,17,18,19]. In a clinical study, sialylation of LOS was associated with an increase in the pathogenic potential, including the development of post-infectious sequelae such as GBS [20]. 

The trillions of microorganisms residing in the gastrointestinal tract form the microbiome, and hold one of the most densely inhabited ecosystems known [21]. The microbiome provides several benefits to the host, including immune maturation, vitamin production, colonization resistance directed against invading pathogens, and the extraction of indigestible carbohydrates from the diet [22]. An essential challenge to the innate immunity is discriminating pathogens from commensal microbes and remaining immunologically tolerant towards the latter. The mechanisms behind this immune tolerance are still not completely understood, and are currently under investigation [23]. An inappropriate activation of TLR4 can result in exaggerated inflammation, gut injury and, in worst case, septic shock, whereas mice lacking TLR4 have been shown not to respond to LPS with septic shock [24]. Nevertheless, TLR4 is required for the elimination of Gram-negative bacteria, but it is believed that the extent and duration of the pro-inflammatory mediator secretion can become harmful to the host [10]. Recognition of commensal bacteria by TLR4 is believed to maintain the intestinal homeostasis and prevent uncontrolled inflammation, but specific microbiota-derived molecular mediators recognized by TLR4 are still largely unknown [23,25]. 

Despite the worldwide rising prevalence of human campylobacteriosis, knowledge regarding the molecular mechanisms underlying pathogen–host interactions is still limited. For a long time, *Campylobacter* infection experiments have been hampered by the lack of appropriate experimental in vivo models [26]. This is mainly due to the physiological colonization resistance exerted by the host-specific gut microbiota composition, preventing conventional laboratory mice, for instance, from *C. jejuni* infection [19]. Upon modification of the gut microbiota, however, the colonization resistance could be overcome, given that *C. jejuni* stably established in the gastrointestinal tract of mice that had been subjected to broad-spectrum antibiotic treatment, which also held true upon reintroduction of a complex gut microbiota derived from human as opposed to murine donors by fecal microbiota transplantation (FMT) [19]. Furthermore, mice have been shown to be about 10,000 times more resistant against TLR4 ligands, such as LPS and LOS, as compared to humans or birds [27,28,29]. Lack of the *il10* gene rendered mice susceptible to LOS and LPS, however [15,30]. Following peroral *C. jejuni* infection, secondary abiotic IL10^-/-^ mice could not only be stably colonized by the pathogen, but also developed acute enterocolitis, mimicking key symptoms of acute campylobacteriosis, such as wasting and bloody diarrhea, within a week post-infection [15,31]. 

Given the predominance of *C. jejuni* causing human campylobacteriosis, research on *C. coli*–host interactions has been neglected to date [32]. Furthermore, the clinical symptoms in the course of human *C. coli* and *C. jejuni* infections can hardly be distinguished. In this study, we therefore investigated the triangle crosstalk (“Ménage à trois”) between the pathogen *C. coli*, the vertebrate host immunity, and, as third component, the human gut microbiota, applying the clinical campylobacteriosis model using secondary abiotic IL10^-/-^ mice. Therefore, we surveyed the pathogen colonization capacity, the microbiota composition before and after infection, and the macroscopic and microscopic inflammatory sequelae in intestinal and systemic compartments following peroral *C. coli* application, of human microbiota-associated (hma) TLR4-deficient IL10^-/-^ mice and IL10^-/-^ mice as controls.

## 2. Results

### 2.1. Gastrointestinal Pathogen Loads Following Peroral C. coli Infection of Human Microbiota-Associated TLR4-Deficient IL10^-/-^ Mice 

**Figure 1:**
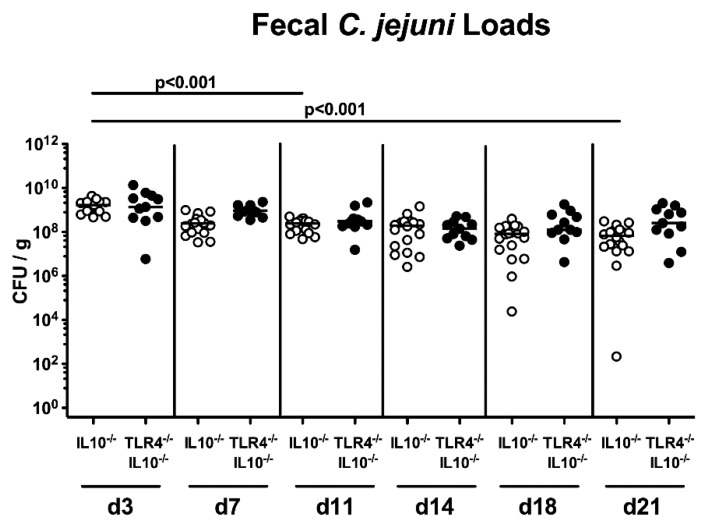
Fecal shedding over time following peroral *Campylobacter coli* infection of Toll-like Receptor-4 (TLR4)-deficient interleukin-10-deficient (IL10^-/-^) mice with a human gut microbiota. Secondary abiotic IL10^-/-^ mice (open circles; n = 18) and TLR4-deficient IL10^-/-^ mice (TLR4^-/-^ IL10^-/-^; closed circles; n = 11) were perorally transplanted with a gut microbiota derived from human fecal donors on day (d) -7, d-6 and d-5. On d0 and d1, human microbiota-associated mice were perorally infected with a *C. coli* patient isolate by gavage, and the pathogen loads were quantitatively enumerated in fecal samples derived over time post-infection by culture (in colony forming units per g; CFU/g). Medians (black bars) and levels of significance (*p*-values) calculated by the Kruskal–Wallis test and Dunn’s post-correction are indicated. Data were pooled from three independent experiments.

**Figure 2:**
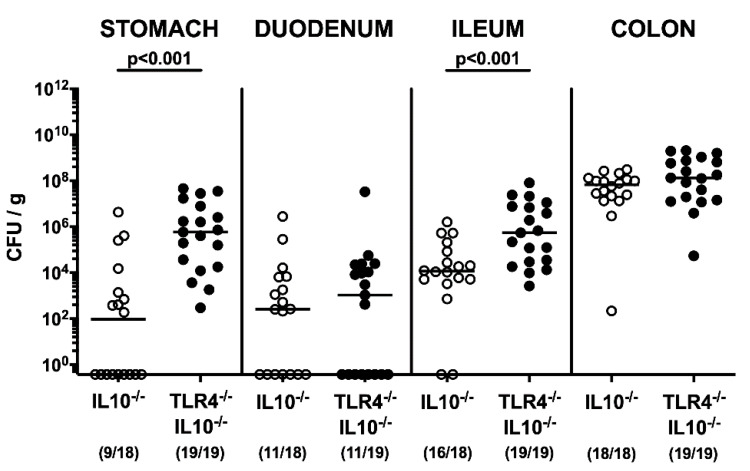
Gastrointestinal *C. coli* burdens, following peroral *C. coli* infection of TLR4-deficient IL10^-/-^ mice with a human gut microbiota. IL10^-/-^ mice (open circles) and TLR4-deficient IL10^-/-^ mice (TLR4^-/-^ IL10^-/-^; closed circles) transplanted with a human gut microbiota were perorally infected with a *C. coli* patient isolate on days 0 and 1 by gavage. Upon necropsy on day 21 post-infection, luminal bacterial loads were enumerated in distinct gastrointestinal compartments by culture (in colony forming units per g; CFU/g) as indicated. Medians (black bars) and numbers of culture-positive mice out of the total number of included animals (in parentheses) in addition to significance levels (*p*-values) calculated by the Mann–Whitney U test are given. Data were pooled from three independent experiments.

Secondary abiotic TLR4-deficient IL10^-/-^ mice and IL10^-/-^ counterparts were associated with a complex human gut microbiota by peroral FMT from human donors, on days -7, -6 and -5 (Figure S1). Following infection of hma mice with 10^8^ viable *C. coli* cells by gavage on days 0 and 1, the pathogen could stably colonize the intestines of mice in a TLR4-independent manner, as indicated by high median loads of more than 10^8^ colony forming units (CFU) per g feces, in both TLR4^-/-^ IL10^-/-^ and IL10^-/-^ mice, until the end of the observation period at day 21 post-infection (Figure 1). At days 11 and 21 following infection of IL10^-/-^, but not of TLR4-deficient IL10^-/-^ mice, approximately one order of magnitude lower fecal *C. coli* burdens could be observed, as compared to day 3 p.i. (*p* < 0.001; Figure 1). Upon necropsy, we further surveyed the pathogen loads in distinct parts of the gastrointestinal tract, and detected comparable *C. coli* loads in the colon and duodenum of mice of either genotype, whereas *C. coli* numbers were higher in the stomach and ileum of TLR4^-/-^ IL10^-/-^ mice as compared to IL10^-/-^ mice at day 21 p.i. (*p* < 0.001; Figure 2). Hence, *C. coli* could stably establish within the gastrointestinal tract of hma TLR4-deficient IL10^-/-^ mice until 21 days post-infection.

### 2.2. Commensal Gut Microbiota Changes Following C. coli Infection of Human Microbiota-Associated TLR4-Deficient IL10^-/-^ Mice

**Figure 3:**
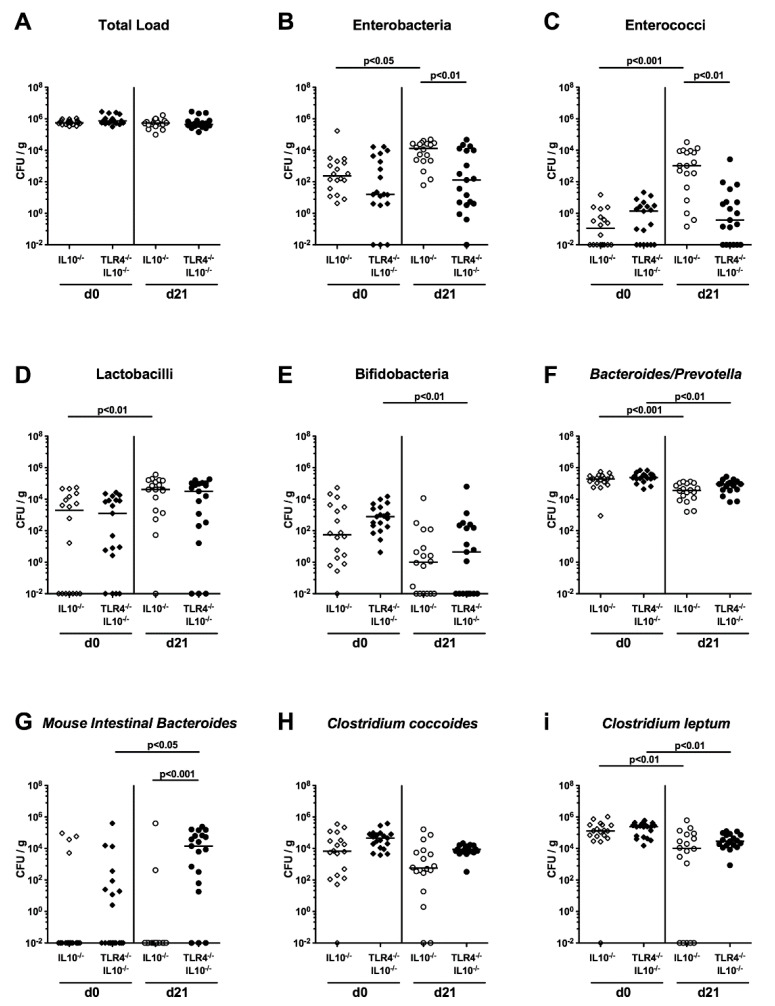
Fecal gut microbiota composition prior and post *C. coli* infection of TLR4-deficient IL10^-/-^ mice with a human gut microbiota. IL10^-/-^ mice (open circles; n = 18) and TLR4-deficient IL10^-/-^ mice (TLR4^-/-^ IL10^-/-^; closed circles; n = 19) harboring a human gut microbiota were perorally infected with a *C. coli* patient isolate on day (d) 0 and d1 by gavage. Immediately before (i.e., d0) and on d21 post-infection, the fecal microbiota composition was determined by quantitative 16S rRNA based Real-Time PCR, and expressed as copies per ng DNA: (**A**) Total eubacterial load; (**B**) enterobacteria; (**C**) enterococci; (**D**) lactobacilli; (**E**) bifidobacteria; (**F**) *Bacteroides/Prevotella* species; (**G**) *Mouse Intestinal Bacteroides*; (**H**) *Clostridium coccoides* group; (**I**) *Clostridium leptum* group. Medians (black bars) and levels of significance (*p*-values) assessed by the Kruskal–Wallis test and Dunn’s post-correction are indicated. Shown data were pooled from three independent experiments.

Within seven days following the first peroral human FMT, the microbiota had stably and comparably established in TLR4-deficient IL10^-/-^ mice and IL10^-/-^ counterparts (i.e., on day 0; not significant (n.s.); Figure 3). We further addressed fecal microbiota changes during *C. coli* infection within each cohort. In mice of either genotype, slightly lower gene numbers for *Bacteroides/Prevotella* spp. and *Clostridium leptum* group could be measured at day 21 p.i., as compared to day 0 (*p* < 0.01–0.001; Figure 3F,I), whereas in IL10^-/-^ mice only, enterobacteria, enterococci and lactobacilli increased in fecal samples during *C. coli* infection (*p* < 0.05–0.001; Figure 3B–D). At day 21 following *C. coli* infection, fecal loads of enterobacteria and enterococci were lower in the feces of TLR4-deficient IL10^-/-^ mice as compared to IL10^-/-^ counterparts (*p* < 0.01; Figure 3B,C). Hence, overall, the fecal microbiota composition of both, the TLR4-deficient IL10^-/-^ hma mice and the IL10^-/-^ hma counterparts were virtually comparable in the naive state, and differentially changed in the course of *C. coli* infection. 

### 2.3. Kinetic Survey of Clinical Signs Exerted by C. coli-Infected Human Microbiota-Associated TLR4-Deficient IL10^-/-^ Mice

**Figure 4:**
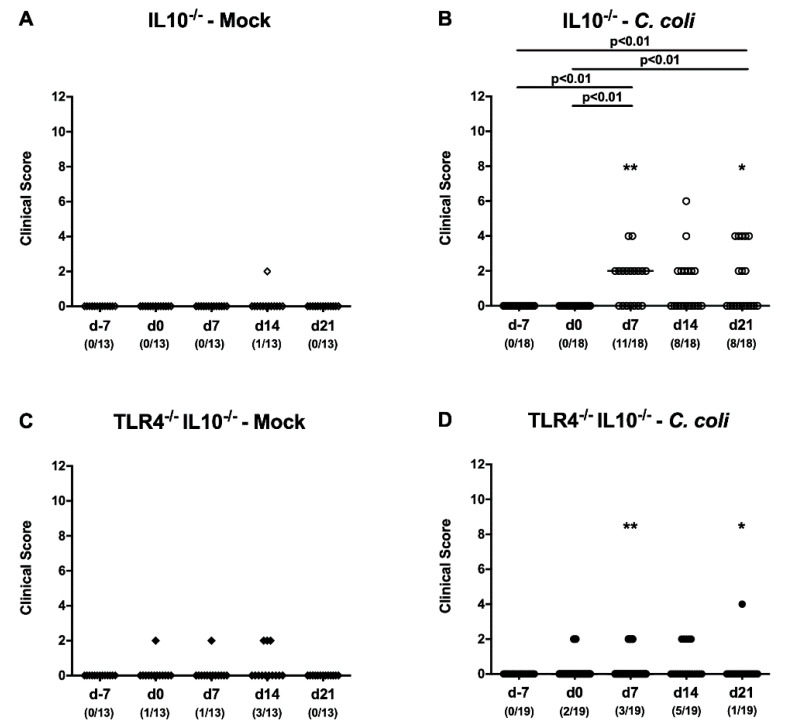
Clinical conditions over time following peroral *C. coli* infection of TLR4-deficient IL10^-/-^ mice with a human gut microbiota. Secondary abiotic IL10^-/-^ mice (**A**, **B**; open symbols) and TLR4-deficient IL10^-/-^ mice (**C**, **D**; TLR4^-/-^ IL10^-/-^; closed symbols) underwent peroral fecal microbiota transplantation on day (d) -7, d-6 and d-5, and were either perorally infected with a *C. coli* (**B**, **D**; circles) patient isolate or received vehicle (**A**, **C**; mock; diamonds) on d0 and d1 by gavage. The overall clinical conditions were monitored applying a standardized clinical scoring system. Medians (black bars) and numbers of mice with a positive clinical score out of the total number of sampled animals (in parentheses), as well as significance levels (*p*-values) assessed by the Kruskal–Wallis test and Dunn’s post-correction, are given. Asterisks indicate significant differences of clinical scores at identical time points between *C. coli* infected cohorts (Mann–Whitney U test; *, *p* < 0.05; **, *p* < 0.01; **B**, **D**). Data were gathered from three independent experiments.

We further quantitatively assessed clinical signs following *C. coli* infection over time with a standardized clinical scoring system addressing characteristic symptoms of human campylobacteriosis, such as wasting and bloody diarrhea. Whereas mock treated hma mice of either genotype were rather uncompromised (Figure 4A,C), hma IL10^-/-^, as opposed to TLR4-deficient IL10^-/-^, mice displayed elevated clinical scores on days 7 and 21 following *C. coli* infection (*p* < 0.01 versus d7 and d0; *p* < 0.05–0.01 versus TLR4^-/-^ IL10^-/-^; Figure 4B,D). When focusing on the abundance of blood in fecal samples, 61.1% of IL10^-/-^ mice, but only 15.8% of TLR4^-/-^ IL10^-/-^, were fecal blood-positive on day 7 p.i., which held true for 44.1% of cases in the former, but only 5.3% of cases in the latter, at day 21 p.i. (Figure S2B,D). Hence, upon peroral infection of hma IL10^-/-^ mice, *C. coli* induced clinical signs in a TLR4-dependent fashion.

### 2.4. Colonic Apoptotic and Immune Cell Responses Following C. coli Infection of Human Microbiota-Associated TLR4-Deficient IL10^-/-^ Mice 

**Figure 5:**
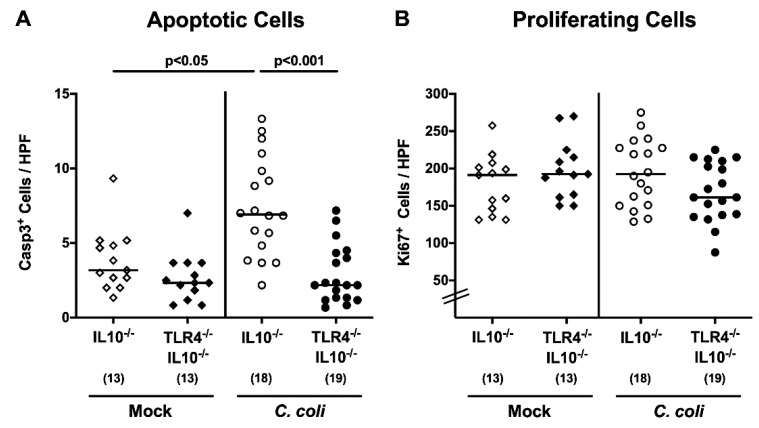
Apoptotic and proliferating colonic epithelial cell responses following peroral *C. coli* infection of TLR4-deficient IL10^-/-^ mice with a human gut microbiota. IL10^-/-^ mice (open symbols) and TLR4-deficient IL10^-/-^ mice (TLR4^-/-^ IL10^-/-^; closed symbols) transplanted with a human gut microbiota were either perorally infected with *C. coli* (circles), or received vehicle (mock; diamonds) on days 0 and 1 by gavage. Upon necropsy on day 21 post-infection, the average counts of epithelial (**A**) apoptotic (Casp3^+^) and (**B**) proliferating (Ki67^+^) cells were microscopically determined in six high power fields (HPF, 400 times magnification) per mouse, in immunohistochemically stained colonic paraffin sections. Medians (black bars) and numbers of examined mice (in parentheses), in addition to levels of significance (*p*-values) calculated by the Kruskal–Wallis test and Dunn’s post-correction or the one-way ANOVA test and Tukey’s post-correction, are indicated. Data were derived from three independent experiments.

**Figure 6:**
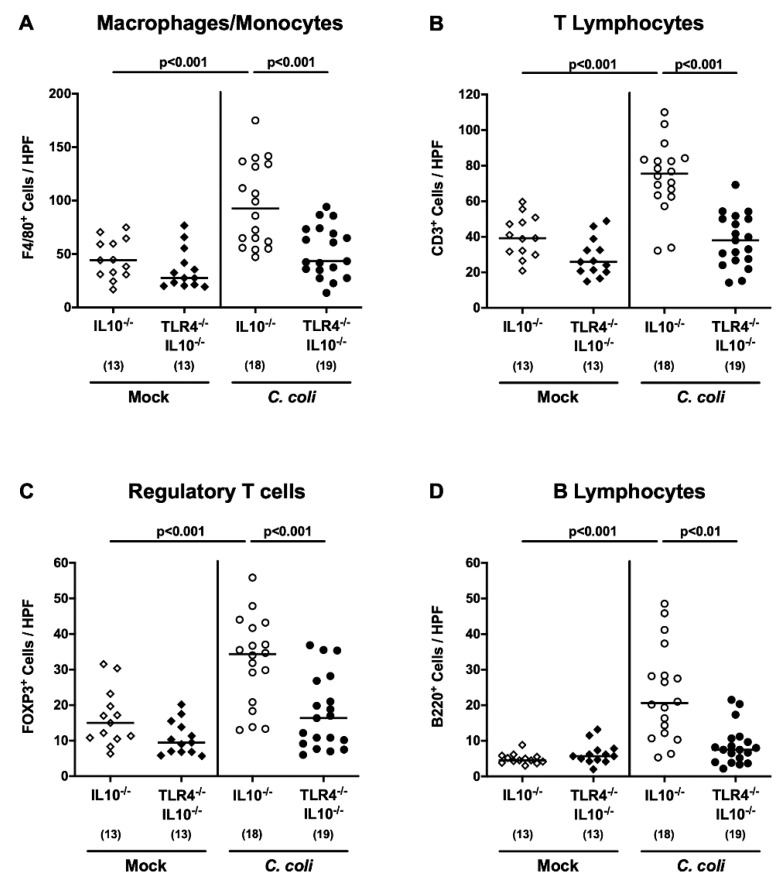
Colonic innate and adaptive immune cell responses following peroral *C. coli* infection of human microbiota-associated TLR4-deficient IL10^-/-^ mice. IL10^-/-^ mice (open symbols) and TLR4-deficient IL10^-/-^ mice (TLR4^-/-^ IL10^-/-^; closed symbols) with a human gut microbiota were either perorally infected with *C. coli* (circles) or received vehicle (mock; diamonds) on days 0 and 1 by gavage. Upon necropsy on day 21 post-infection, the average counts of (**A**) macrophages and monocytes (F4/80^+^), (**B**) T lymphocytes (CD3^+^), (**C**) regulatory T cells (FOXP3+) and (**D**) B lymphocytes (B220+) were microscopically determined in six high power fields (HPF, 400 times magnification) per mouse in immunohistochemically stained colonic paraffin sections. Medians (black bars) and numbers of examined mice (in parentheses), as well as levels of significance (*p*-values) determined by the one-way ANOVA test and Tukey’s post-correction or the Kruskal–Wallis test and Dunn’s post-correction, are shown. Data were pooled from three independent experiments.

We next assessed *C. coli*-induced inflammatory immune responses in TLR4-deficient IL10^-/-^ hma mice. Apoptosis is considered to be a reliable marker for the grading of intestinal inflammatory conditions [19], and we therefore quantitated apoptotic colonic epithelial cell numbers by in situ immunohistochemical staining of large intestinal paraffin sections, taken upon necropsy, with an anti-cleaved caspase3 antibody. At day 21 following *C. coli* infection, caspase3^+^ colonic epithelial cell numbers were higher in IL10^-/-^, as compared to both TLR4-deficient IL10^-/-^ mice and mock treated IL10^-/-^ controls (*p* < 0.001 and *p* < 0.05, respectively; Figure 5A). Numbers of Ki67^+^ colonic epithelial cells, indicative of cell proliferation and regeneration, however, were comparable between either cohort (n.s.; Figure 5B). We further quantitated colonic immune cell responses, again by in situ immunohistochemical staining of large intestinal paraffin sections, against F4/80+, in order to detect innate immune cell subsets such as macrophages and monocytes, and against CD3, FOXP3 and B220, to survey adaptive immune cell populations, including T lymphocytes, regulatory T cells and B lymphocytes, respectively. Similar to apoptotic epithelial cells, respective immune cell subsets increased in the colonic mucosa and lamina propria, until day 21 following *C. coli* infection, of IL10^-/-^ but not TLR4-deficient IL10^-/-^ mice (*p* < 0.01–0.001; Figure 6). Hence, *C. coli* TLR4 dependently induced apoptotic epithelial and innate, as well as adaptive, immune responses in the large intestines of hma IL10^-/-^ mice.

### 2.5. Intestinal and Systemic Pro-Inflammatory Cytokine Secretion Following C. coli Infection of Human Microbiota-Associated TLR4-Deficient IL10^-/-^ Mice 

**Figure 7:**
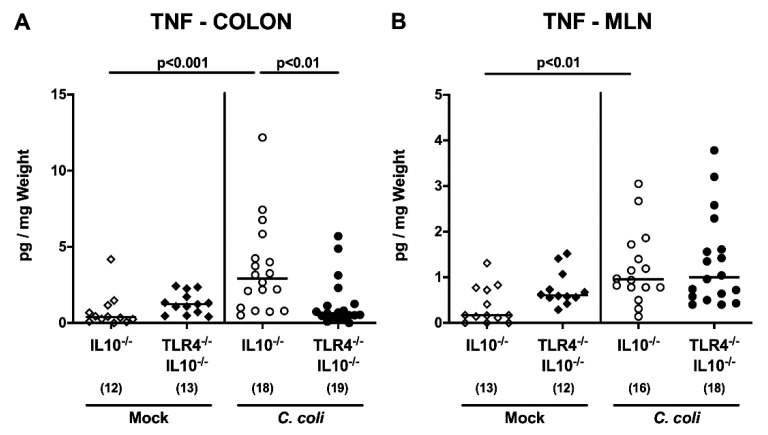
Intestinal TNF secretion following peroral *C. coli* infection of TLR4-deficient IL10^-/-^ mice transplanted with a human gut microbiota. IL10^-/-^ mice (open symbols) and TLR4-deficient IL10^-/-^ mice (TLR4^-/-^ IL10^-/-^; closed symbols) associated with a human gut microbiota were either perorally infected with *C. coli* (circles) or received vehicle (mock; diamonds) on days 0 and 1 by gavage. Upon necropsy on day 21 post-infection, TNF concentrations were determined in ex vivo biopsies taken from (**A**) colon and (**B**) mesenteric lymph nodes (MLN). Medians (black bars) and numbers of sampled mice (in parentheses), as well as significance levels (*p*-values) determined by the one-way ANOVA test and Tukey’s post-correction or the Kruskal–Wallis test and Dunn’s post-correction, are illustrated. Definite outliers were removed after being identified by the Grubb’s test (α = 0.001). Data were pooled from three independent experiments.

**Figure 8:**
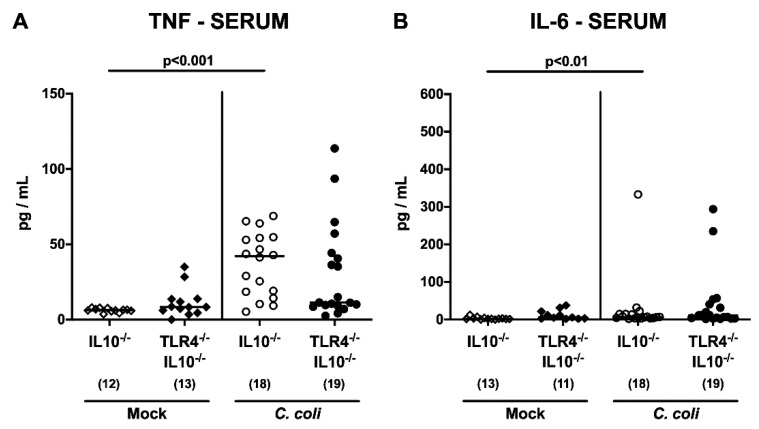
Systemic pro-inflammatory cytokine secretion following peroral *C. coli* infection of human microbiota-associated TLR4-deficient IL10^-/-^ mice. IL10^-/-^ mice (open symbols) and TLR4-deficient IL10^-/-^ mice (TLR4^-/-^ IL10^-/-^; closed symbols) with a human gut microbiota were either perorally infected with *C. coli* (circles) or received vehicle (mock; diamonds) on days 0 and 1 by gavage. (**A**) TNF and (**B**) IL-6 concentrations were quantified in serum samples taken upon necropsy on day 21 post-infection. Medians (black bars) and numbers of analyzed animals (in parentheses), in addition to significance levels (*p*-values) determined by the one-way ANOVA test and Tukey’s post-correction or the Kruskal–Wallis test and Dunn’s post-correction, are indicated. Definite outliers were removed after being identified by the Grubb’s test (α = 0.001). Data were pooled from three independent experiments.

We next addressed whether *C. coli*-induced, pro-inflammatory cytokine secretion in hma IL10^-/-^ mice also occurred in a TLR4-dependent manner. At day 21 p.i., higher tumor necrosis factor (TNF) concentrations could be measured in ex vivo biopsies taken from colon and mesenteric lymph nodes (MLN) of IL10^-/-^, but not TLR4-deficient IL10^-/-^, mice, as compared to mock treated counterparts (*p* < 0.001 and *p* < 0.05, respectively; Figure 7). Strikingly, the TLR4-dependent, *C. coli*-induced, pro-inflammatory cytokine secretion was not restricted to the intestinal tract, but could also be detected systemically, given that higher TNF and interleukin-6 (IL-6) concentrations were measured in serum samples taken from *C. coli* infected as compared to mock control IL10^-/-^, but not TLR4^-/-^ IL10^-/-^, mice upon necropsy (*p* < 0.001 and *p* < 0.01, respectively; Figure 8). Hence, upon peroral infection of hma IL10^-/-^ mice, *C. coli* induced the secretion of pro-inflammatory cytokines not only in the intestinal tract, but also systemically, in a TLR4-dependent fashion.

## 3. Discussion

Research on *C. coli* has been widely neglected to the present date, although the pathogen is the second most common causative agent of human *Campylobacter* infections after *C. jejuni* [33]. Despite the fact that several studies point towards genetic differences between *C. coli* and *C. jejuni*, knowledge regarding the phenotypic differences is virtually lacking [34,35,36]. Very recently, our group demonstrated that, in contrast to *C. jejuni*, a *C. coli* patient isolate could override the colonization resistance of conventionally colonized wildtype mice, and remained in the large intestine up to three weeks post-infection [37]. In the present study, we aimed at investigating possible TLR4-dependent immune responses to the same *C. coli* strain in secondary abiotic IL10^-/-^ mice harboring a human gut microbiota. Therefore, secondary abiotic IL10^-/-^, and TLR4^-/-^ IL10^-/-^, mice were subjected to peroral human FMT on three consecutive days, one week prior to infection. The quantification of the most prominent intestinal bacterial genera by molecular microbiota analyses revealed that, until the day of the first infection, the human gut microbiota had established in both IL10^-/-^ and TLR4^-/-^ IL10^-/-^ mice. Up to three weeks post-challenge, *C. coli* remained in the gastrointestinal tract of mice of both genotypes at high loads. While in the colon and duodenum the luminal *C. coli* loads were comparable, the pathogen burdens were approximately two and even four log orders of magnitude higher in the ileum and stomach, respectively, in TLR4-deficient IL10^-/-^ as compared to IL10^-/-^ counterparts, on day 21 post-infection. Whereas TLR4 expression has been demonstrated along the entire gastrointestinal tract [38], its expression pattern might differ between distinct gastrointestinal compartments. In the large intestines of healthy mice, for instance, TLR4 was shown to be mainly expressed at the basolateral side of the epithelium indicative for a spatial regulation to avoid constant activation from luminal bacteria. In the terminal ileum, however, TLR4 was rather apically expressed [38]. Apical and basolateral TLR4 expression was shown in the stomach of infected mice that had been infected with *Helicobacter pylori*, which are genetically closely related to *Campylobacter* species [39]. It is tempting to speculate that basolateral expression of TLR4 allows the pathogen to colonize at higher loads than apical TLR4 expression does.

Three weeks following peroral *C. coli* infection, TLR4-deficient IL10^-/-^ mice were clinically less compromised, and tested positive less frequently for fecal blood, as compared to infected IL10^-/-^ counterparts. Of note, irrespective of whether *C. coli*-infected or not, clinical scores were comparable in TLR4-deficient IL10^-/-^ mice. Furthermore, *C. coli* induced apoptotic responses in colonic epithelia in a TLR4-dependent manner, whereas colonic epithelial cell proliferative/regenerative measures, counteracting potential pathogen-induced cell damage, were comparable in *C. coli* and mock challenged mice of either genotype. In support, TLR4 has been previously shown to act as a potent inducer of cell apoptosis [15,19,40]. 

Moreover, innate and adaptive pro-inflammatory immune responses, that could be assessed in the intestinal tract on day 21 following *C. coli* infection of IL10^-/-^ hma mice, were TLR4-dependent. Remarkably, the TLR4-dependently induced immune responses upon *C. coli* infection were not limited to the intestinal tract, but were also effective systemically, given that only in IL10^-/-^, but not TLR4-deficient IL10^-/-^, mice, increased serum concentrations of pro-inflammatory cytokines, such as TNF and IL-6, could be measured at day 21 following *C. coli* challenge. We were not able to culture viable *C. coli* that might potentially have translocated from the intestines to the extra-intestinal, including systemic, compartments. It is, however, highly likely that soluble pathogenic cell wall constituents, such as LOS, were responsible for the observed systemic pro-inflammatory effects, and that viable bacteria might have already been cleared by the immune system as late as 21 days post-infection. 

The observed TLR4-mediated inflammatory effects following peroral *Campylobacter* challenge are supported by our previous studies applying different murine *C. jejuni* infection and inflammation models. In fact, *C. jejuni* induced intestinal pro-inflammatory immune responses TLR4-dependently in secondary abiotic wildtype mice on day 14 post-infection [19], which also held true for secondary abiotic IL10^-/-^ mice suffering from acute enterocolitis on day 6 upon *C. jejuni* challenge [15]. Of note, this is the first report about TLR4-dependent immune responses in *Campylobacter* infected mice with a human gut microbiota. While the microbiota composition was virtually comparable before infection in hma mice of either genotype, three weeks later, hma TLR4-deficient IL10^-/-^ mice harbored approximately four orders of magnitude higher *Mouse Intestinal Bacteroides* gene numbers in their feces as compared to infected IL10^-/-^ counterparts. It is tempting to speculate that the TLR4^-/-^ IL10^-/-^ mice were more engaged in coprophagy. *Mouse Intestinal Bacteroides* are most commonly found in the intestine of mice, but can also be found at low levels in humans [41]. Of interest, during *C. coli* infection, the enterobacterial loads increased by approximately two orders of magnitude in the feces derived from IL10^-/-^, but not TLR4-deficient IL10^-/-^, hma mice. We have previously shown that acute and chronic inflammation in the small and large intestines are accompanied by gut microbiota shifts towards commensal Gram-negative bacterial species, including enterobacteria such as *E. coli*, overgrowing the intestinal lumen and further perpetuating the inflammatory scenario [42,43,44,45,46,47,48,49,50]. Hence, the observed increased enterobacterial loads in *C. coli*-infected hma IL10^-/-^ mice, as opposed to TLR4-deficient IL10^-/-^ mice, parallels the pro-inflammatory immune responses in the former but not the latter. Furthermore, gut microbiota changes, that were associated with increased enterobacterial loads due to inflammatory conditions or enterobacterial feeding, facilitated *C. jejuni* infection [26,49,51,52,53]. In the present study, however, enterobacterial loads were comparable in hma mice of either genotype, resulting in comparable *C. coli* colonization densities in the large intestines. 

Nevertheless, one should be cautious of drawing conclusions regarding potential TLR4-dependent microbiota changes in *C. coli*-infected hma IL10^-/-^ mice based upon the presented data, given that the ”humanized” mouse model has its limitations, as any other experimental model. Among those, genetic, physical and environmental factors have to be taken into consideration. The interactions between host and microbiome have been shaped by co-evolution, and are considered crucial for proper development of the immune system, nutrient utilization, and for mechanisms such as colonization resistance [54]. It is challenging to ensure persistence of the human microbiota in the murine host over time, when mice are not supplemented with a human diet or further human fecal transplantations. It should also be considered that certain bacterial species are lost when freezing the human fecal donor samples, or that certain species colonize the gut in a host-specific manner. Nevertheless, despite these limitations, mice can be transplanted with a human gut microbiota surprisingly well, and are informative models for unravelling interactions between pathogens, microbiota and the host immune system, as shown previously [19,50,53,55,56,57,58,59,60,61]. Furthermore, it is virtually impossible to exactly dissect whether the observed TLR4-dependent immune responses, in *C. coli*-infected hma IL10^-/-^ mice, where solely due to TLR4-dependent signaling of pathogenic *Campylobacter*-related and/or commensal gut bacterial factors.

In conclusion, this is the first study demonstrating that TLR4 is required for mediating intestinal and systemic pro-inflammatory immune responses upon *C. coli* infection of IL10^-/-^ mice harboring a human gut microbiota. Based upon these findings, and given the major impact of *C. jejuni* LOS during initiation and perpetuation of campylobacteriosis, it is likely that pathogenic LOS might also play an integral role during *C. coli* infection. Future studies need to address whether the observed TLR4-dependent inflammatory responses are affected by the microbiota composition. It is tempting to speculate that certain microbiota-derived compounds could protect, or at least dampen, the inflammatory responses induced by *C. coli* TLR4 ligands.

## 4. Materials and Methods 

### 4.1. Ethics Statement

The animal experiments were carried out in agreement with the European Guidelines for animal welfare (2010/63/EU), and approved by the commission for animal experiments headed by the “Landesamt für Gesundheit und Soziales” (LaGeSo, Berlin, Germany, registration numbers G0172/16 and G0247/16). The clinical conditions of mice were surveyed twice a day.

### 4.2. Generation of Secondary Abiotic Mice

Age- and sex-matched, TLR4-deficient IL10^-/-^ (TLR4^-/-^ IL10^-/-^) mice and IL10^-/-^ mice (all in C57BL/10 background) were bred and maintained in groups of 2 to 5 individuals under specific pathogen-free (SPF) and standardized conditions (22–24 °C room temperature, 55% ± 15% humidity, 12 h light / 12 h dark cycle) in the same unit of the Forschungseinrichtungen für Experimentelle Medizin (FEM, Charité - University Medicine Berlin, Berlin, Germany). Mice were kept in cages covered by filter tops within an experimental semi-barrier (only accessible with lab coat, shoe covers, hair net, medical mask and sterile gloves for the investigators) and had free access to autoclaved standard chow (food pellets: sniff R/M-H, V1534-300, Sniff, Soest, Germany).

Secondary abiotic mice with a virtually depleted microbiota [62] were generated by transferring 3-week old littermate mice into sterile cages and subsequently treating them with a broad-spectrum antibiotic cocktail for eight weeks, by adding ampicillin plus sulbactam (1 g/L; Ratiopharm, Ulm, Germany), vancomycin (500 mg/L; Cell Pharm, Hannover, Germany), ciprofloxacin (200 mg/L; Bayer Vital, Leverkusen, Germany), imipenem/cilastatin (250 mg/L; Fresenius Kabi, Bad Homburg, Germany) and metronidazole (1 g/L; B. Braun, Melsungen, Germany) to the drinking water (ad libitum) [42]. Throughout the experiment, mice were retained in a sterile environment (autoclaved food and drinking water) and managed under strict aseptic conditions to avoid contaminations.

### 4.3. Reassociation of Secondary Abiotic Mice with a Human Gut Microbiota by Fecal Microbiota Transplantation

Three days prior reassociation of secondary abiotic mice with a complex human intestinal microbiota, complete antibiotic washout was ensured by replacing the antibiotic cocktail with autoclaved tap water (ad libitum). Fresh fecal samples free of enteropathogenic bacteria, viruses and parasites were voluntarily donated from five healthy individuals, dissolved in sterile phosphate buffered saline (PBS; Thermo Fisher Scientific, Waltham, MA, USA), aliquoted, and stored at −80 °C as described before [19,63]. Immediately before the human FMT, individual fecal aliquots were thawed and pooled. The main bacterial groups within the donor suspension were quantitated by cultural and molecular methods as described previously [19,55]. Hma mice were generated by transplanting secondary abiotic animals with 0.3 mL of the donor suspension by gavage on three consecutive days (i.e, days -7, -6, -5). The abundance of bacterial groups differed less than 0.5 log orders of magnitude among independent experiments. To ensure that the human microbiota had properly established in the murine host, mice were subjected to *C. coli* infection seven days after the initial human FMT. Immediately before the first infection and upon necropsy (i.e., day 21 post-infection) individual fecal samples were taken for quantitative cultural and molecular analyses of main intestinal bacterial phylogenetic groups as stated elsewhere [57,60,61].

### 4.4. C. coli Infection and Gastrointestinal Colonization

The *C. coli* strain was initially isolated from a patient displaying bloody diarrhea and kindly provided by Dr. Torsten Semmler (Robert-Koch-Institute Berlin, Berlin, Germany). The pathogen strain was stored at −80 °C. Prior to infection, *C. coli* were freshly cultivated on columbia agar (supplemented with 5% sheep blood) and karmali agar plates (both from Oxoid, Wesel, Germany) after thawing from the stock. On two consecutive days (i.e., days 0 and 1), hma mice were perorally challenged with 10^8^ colony forming units (CFU) of either the *C. coli* patient isolate or received vehicle (i.e., PBS) by gavage. In order to assess intestinal colonization properties, *C. coli* loads were enumerated in fecal samples collected over time post-infection and in luminal samples derived from distinct parts of the gastrointestinal tract (i.e., from the stomach, duodenum, ileum and colon) upon necropsies by culture as stated elsewhere [19,64]. In brief, for *C. coli* quantification, serial dilutions of samples were plated onto columbia agar plates containing 5% sheep blood and karmali agar plates (both from Oxoid, Wesel, Germany) and incubated in a jar under microaerophilic conditions for 48 h at 37 °C. 

### 4.5. Cultural and Culture-Independent (i.e., Molecular) Survey of the Human Donor Suspensions and Gut Microbiota 

For extensive quantitative survey of the microbiota composition in fecal human donor suspensions and large intestinal luminal contents, samples were homogenized in sterile PBS and enumerated from serial dilutions on respective solid media as described previously [42]. Bacteria were grown at 37 °C for at least two days under aerobic, microaerobic and anaerobic conditions as stated elsewhere [42,43,44]. 

For molecular gut microbiota analysis, the total genomic DNA was extracted from the human donor suspension and colonic luminal samples as described previously [42]. Briefly, DNA was quantitated by using Quant-iT PicoGreen reagent (Invitrogen, Carlsbad, CA, USA). The DNA concentration was adjusted to 1 ng per µL. The main bacterial groups abundant in the microbiota of the hma mice were assessed by quantitative real-time polymerase chain reaction (qRT-PCR) with species-, genera- or group-specific 16S rRNA gene primers (Tib MolBiol, Berlin, Germany) as described previously [19,60]. 

### 4.6. Clinical Conditions

Before and after pathogen application, we quantitatively determined the clinical conditions of mice by using a standardized cumulative clinical score on a daily basis. The score reached a maximum of 12 points, addressing the clinical aspect (0: normal; 1: ruffled fur; 2: less locomotion; 3: isolation; 4: severely compromised locomotion, pre-final aspect), the occurrence of blood in feces (0: no blood; 2: microscopic detection of blood by the Guajac method using Haemoccult, Beckman Coulter, Krefeld, Germany; 4: macroscopic blood visible), and the presence of diarrhea (0: formed feces; 2: pasty feces; 4: liquid feces) as described earlier [31]. Fecal blood positivity rates were calculated from the ratio of microscopically (including macroscopically) fecal blood-positive mice to the total number of analyzed animals. 

### 4.7. Sampling Procedures

Groups of mice were sacrificed by isofluran inhalation (Abbott, Chicago, IL, USA) on day 21 post *C. coli* challenge. Ex vivo biopsies from MLN and colon as well as luminal gastrointestinal samples (from stomach, duodenum, ileum and colon) were collected under sterile conditions. For serum cytokine measurements cardiac blood was taken. Colonic samples were derived from each mouse in parallel for microbiological, immunohistopathological and immunological analyses.

### 4.8. Immunohistochemistry

In situ immunohistochemical analyses were performed in large intestinal ex vivo biopsies that had been immediately fixated in 5% formalin and embedded in paraffin as recently reported [47,65,66,67]. Briefly, apoptotic epithelial cells, proliferative epithelial cells, macrophages/monocytes, T lymphocytes, regulatory T cells (Tregs), and B lymphocytes, in 5 μm colonic paraffin sections, were detected by staining the tissue sections with primary antibodies directed against cleaved caspase 3 (Asp175, Cell Signaling, Beverly, MA, USA, 1:200), Ki67 (TEC3, Dako, Glostrup, Denmark, 1:100), F4/80 (# 14-4801, clone BM8, eBioscience, San Diego, CA, USA, 1:50), CD3 (#N1580, Dako, 1:10), FOXP3 (clone FJK-165, #14-5773, eBioscience, 1:100), and B220 (No. 14-0452-81, eBioscience; 1:200), respectively. Positively stained cells were counted by a blinded independent investigator applying light microscopy (magnification 100× and 400×). The average number of respective positively stained cells in each sample was determined within at least six high power fields (HPF, 0.287 mm^2^, 400× magnification).

### 4.9. Pro-Inflammatory Cytokines in Intestinal and Serum Samples

Distal large intestinal ex vivo biopsies (approximately 1 cm^2^ tissue cut longitudinally and washed in PBS) and ex vivo biopsies derived from MLN (3 lymph nodes) were placed in 24 flat-bottom well culture plates (Thermo Fisher Scientific, Waltham, MA, USA) containing 500 μL serum-free RPMI 1640 medium (Gibco, life technologies) supplemented with penicillin (100 U/mL) and streptomycin (100 µg/mL; Biochrom, Berlin, Germany). After incubation for 18 h at 37 °C, respective culture supernatants as well as serum samples were tested for TNF and IL-6 by the Mouse Inflammation Cytometric Bead Assay (CBA; BD Biosciences, Heidelberg, Germany) on a BD FACSCanto II flow cytometer (BD Biosciences). 

### 4.10. Statistical Analysis

Medians and levels of significance were determined with GraphPad Prism v8, USA. The Student’s t test was applied for pairwise comparisons of normally distributed data, whereas the Mann–Whitney test was used for pairwise comparisons of not normally distributed data. For multiple comparisons, the one-sided ANOVA with Tukey post-correction was assigned for normally distributed data, and the Kruskal–Wallis test with Dunn’s post-correction for not normally distributed data. Two-sided probability (*p*) values ≤0.05 were considered significant. Definite outliers were removed after being identified by the Grubb’s test (α = 0.001). Data were pooled from three independent experiments.

## Supplementary Materials

The following are available online at https://www.mdpi.com/2076-0817/9/5/386/s1, Figure S1: Commensal bacterial composition of human donor suspensions used for fecal microbiota transplantation, Figure S2: Abundance of fecal blood over time following peroral *C. coli* infection of TLR4-deficient IL10^-/-^ mice harboring a human gut microbiota.
