# Supplementary material for: Toll-Like Receptor-4 Dependent Intestinal and Systemic Sequelae Following Peroral Campylobacter coli Infection of IL10 Deficient Mice Harboring a Human Gut Microbiota
Source: Pathogens. 2020 May 18;9(5):386. doi: 10.3390/pathogens9050386 (PMC7281621; doi:10.3390/pathogens9050386)
Supplement: Supplementary file 1 [file pathogens-09-00386-s001.pdf]

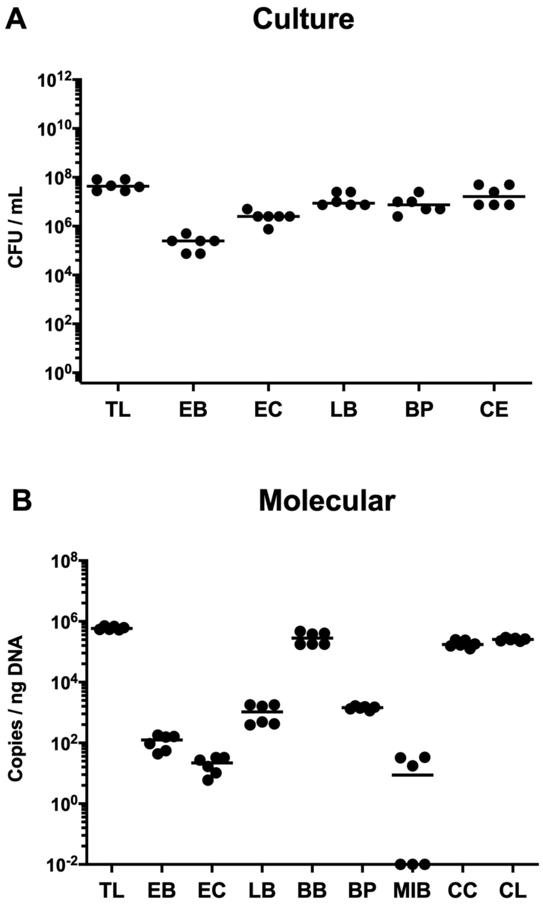

Figure S1. Commensal bacterial composition of human donor suspensions used for fecal microbiota transplantation. Immediately before fecal microbiota composition on three consecutive days (i.e., days -7, -6, -5), the commensal bacterial compositions of human donor suspensions were quantitatively assessed by **(A)** culture and **(B)** culture-independent 16S rRNA based methods and expressed as colony forming units per ml (CFU / ml) and copies / ng DNA, respectively. Medians (black bars) are indicated. Shown data were pooled from two independent experiments. TL: total bacterial load; EB: enterobacteria; EC: enterococci; LB: lactobacilli; BB: bifidobacterial; BP: *Bacteroides* / *Prevotella* species; MIB: *Mouse Intestinal Bacteroides*; CE: *Clostridium* / *Eubacterium* species; CC: *Clostridium coccoides* group; CL: *Clostridium leptum* group.

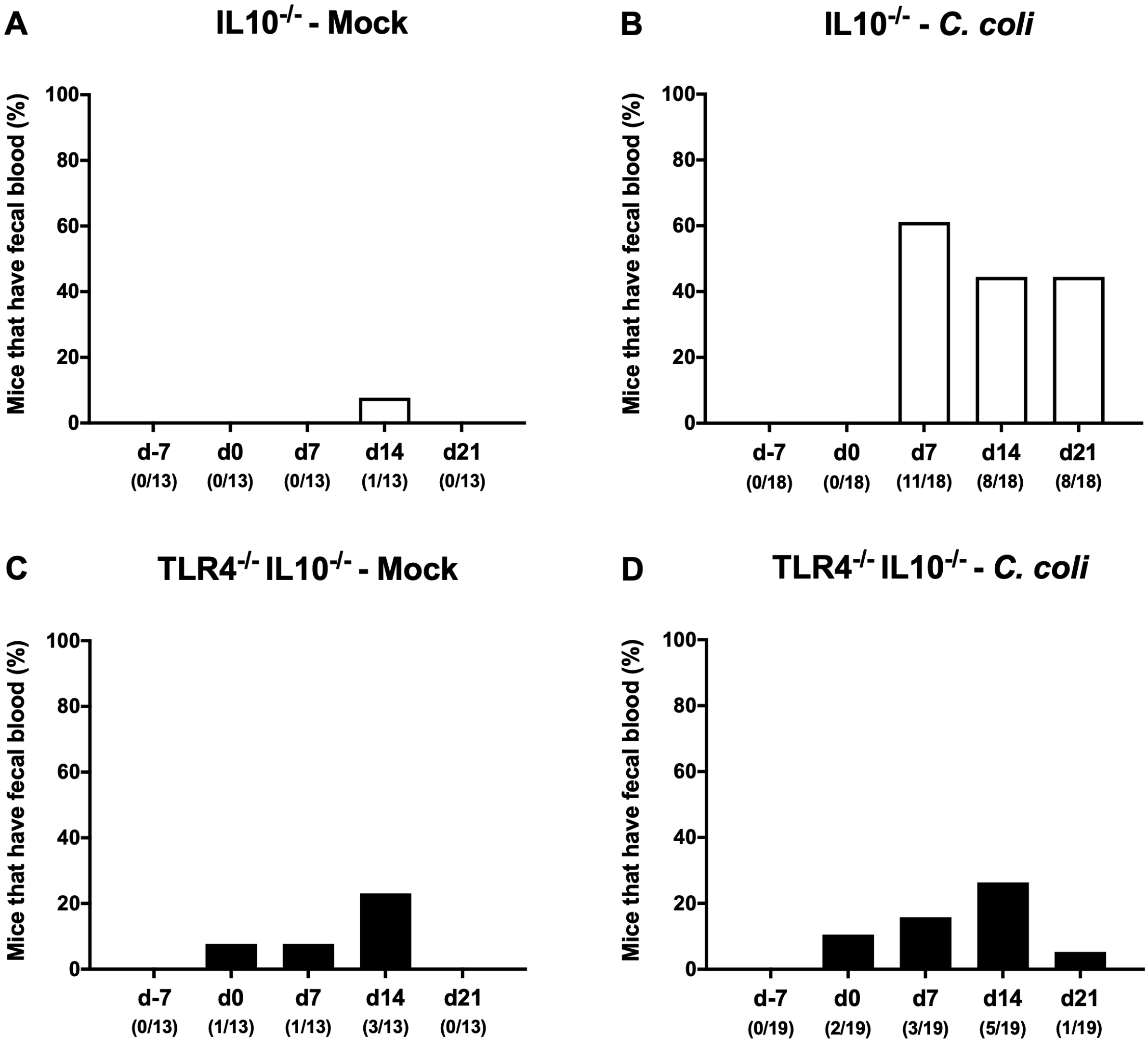

Figure S2: Abundance of fecal blood over time following peroral *C. coli* infection of TLR4 deficient IL10$^{-/-}$ mice harboring a human gut microbiota. Secondary abiotic IL10$^{-/-}$ mice (**A, B**) and TLR4 deficient IL10$^{-/-}$ mice (**C,D;** TLR4$^{-/-}$ IL10$^{-/-}$) were subjected to peroral fecal microbiota transplantation from human donors on day (d) -7, d-6 and d-5 and were either perorally infected with *C. coli* (**B,D**) or received vehicle (**A,C**; mock) on d0 and d1 by gavage. Macroscopic or microscopic detection of fecal blood was surveyed in each mouse over time post-infection. Bars indicate the cumulative frequencies of fecal blood (in %). Numbers of fecal blood positive mice out of the total number of analyzed animals are given in parentheses. Data were pooled from three independent experiments.
